# Wild pollinator activity negatively related to honey bee colony densities in urban context

**DOI:** 10.1371/journal.pone.0222316

**Published:** 2019-09-12

**Authors:** Lise Ropars, Isabelle Dajoz, Colin Fontaine, Audrey Muratet, Benoît Geslin

**Affiliations:** 1 IMBE, Aix Marseille Univ, Avignon Université, CNRS, IRD, Marseille, France; 2 Institut d’Ecologie et des Sciences de l’Environnement de Paris (iEES Paris UMR 7618) Equipe Ecologie et Evolution des réseaux d’interactions, Université Paris Diderot, CNRS-SU, Paris, France; 3 Centre d’Ecologie et des Sciences de la Conservation (CESCO UMR 7204), CNRS-Muséum National d’Histoire Naturelle-SU, Paris, France; 4 Agence Régionale de la Biodiversité en Île-de-France (ARB ÎdF), Paris, France; 5 Laboratoire Image, Ville, Environnement (LIVE UMR 7362), Université de Strasbourg, Strasbourg, France; Universitat Leipzig, GERMANY

## Abstract

As pollinator decline is increasingly reported in natural and agricultural environments, cities are perceived as shelters for pollinators because of low pesticide exposure and high floral diversity throughout the year. This has led to the development of environmental policies supporting pollinators in urban areas. However, policies are often restricted to the promotion of honey bee colony installations, which resulted in a strong increase in apiary numbers in cities. Recently, competition for floral resources between wild pollinators and honey bees has been highlighted in semi-natural contexts, but whether urban beekeeping could impact wild pollinators remains unknown. Here, we show that in the city of Paris (France), wild pollinator visitation rates are negatively correlated to honey bee colony densities present in the surrounding landscape (500m –slope = -0.614; p = 0.001 –and 1000m –slope = -0.489; p = 0.005). Regarding the morphological groups of wild pollinators, large solitary bee and beetle visitation rates were negatively affected by honey bee colony densities within a 500m buffer (slope = -0.425, p = 0.007 and slope = - 0.671, p = 0.002, respectively) and bumblebee visitation rates were negatively affected by honey bee colony density within a 1000m buffer (slope = - 0.451, p = 0.012). Further, lower interaction evenness in plant-pollinator networks was observed with high honey bee colony density within a 1000m buffer (slope = -0.487, p = 0.008). Finally, honey bees tended to focus their foraging activity on managed rather than wild plant species (student t-test, p = 0.001) whereas wild pollinators equally visited managed and wild species. We advocate responsible practices mitigating the introduction of high density of honey bee colonies in urban environments. Further studies are however needed to deepen our knowledge about the potential negative interactions between wild and domesticated pollinators.

## Introduction

The recent decline of pollinating insect populations is driven by a conjunction of factors, including habitat fragmentation, use of pesticides, multiplication of pathogens, global warming and the decline of the wild flora [1]. Agricultural landscapes have changed, harbouring fewer floral resources and habitats to support diverse pollinating communities [2,3]. Consequently, many agricultural landscapes are becoming less conducive for pollinators and for beekeeping activities [4]. At the same time, areas that were previously rarely exploited by beekeepers are now under a strong pressure to receive apiaries; this is the case in natural habitats and cities [5,6]. Indeed, cities harbour diverse plant species flourishing all year long due to management practices [7] and heat island effect, thus providing resources throughout the year for pollinators [8]. The low pesticide policies applied in many conurbations may also create favourable conditions for the maintenance of diverse pollinator communities [9]. In the same time, honey bees are perceived as a symbol of biodiversity and ecosystem well-being by many city-dwellers and the media [10]. Many citizens have thus installed colonies as their own contribution to mitigate the pollinator decline [11,12] and urban introductions of honey bee colonies have been promoted by public authorities and decision makers. In many cities, this has translated into very recent and rapid increases in the number of honey bee colonies (e.g. 10 colonies per km^2^ in London–United Kingdom [13], 15 colonies per km^2^ in Brussels–Belgium [14]).

**Fig 1:**
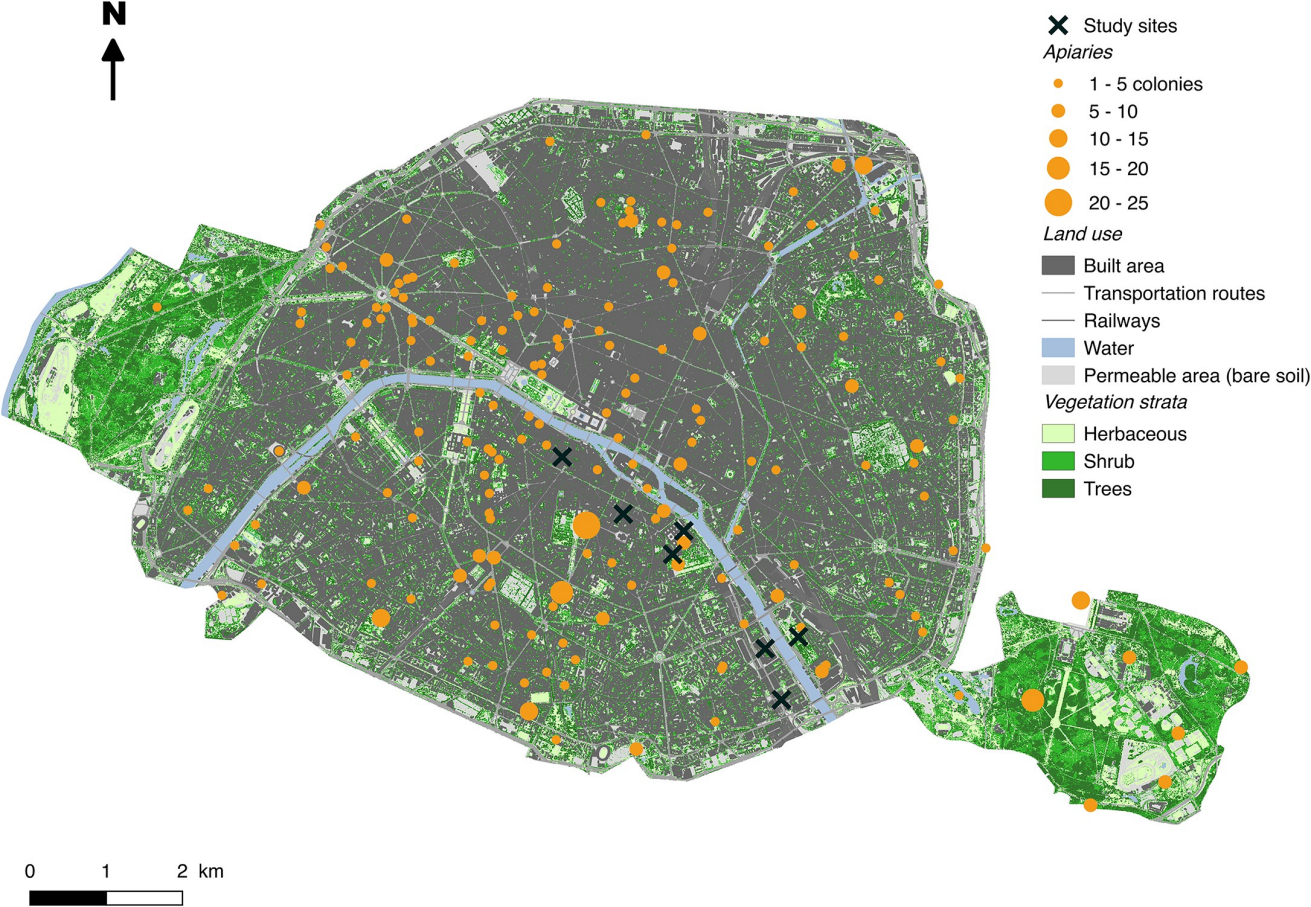
Location of honey bee colonies and study sites in the city of Paris. Vegetation height and land use maps were obtained from APUR database (http://opendata.apur.org/datasets/).

However, cities are not depauperate in wild pollinating insects and there is increasing evidence that they host diverse assemblages of wild bees [15,16]. This has led to rising concern about numerous introductions of honey bees in cities, that may negatively impact the wild pollinating fauna through competition for floral resources [11]. In other habitats, such as semi-natural (calcareous meadows [17] or scrubland [18,19]) or agricultural landscapes, several authors have detected exploitative competition between domesticated and wild pollinators through the monopolization of floral resources by honey bees [20,21]. However, we know of no studies that have assessed that honey bee introductions in cities could impact wild pollinator communities and their foraging activity on urban plant communities. Moreover, the effect of increasing honey bee densities has rarely been assessed using network approaches [11]. Massively introduced honey bees might impair the pollination function at community level by, for example, focusing their visits on managed (ornamental) plant species rather than wild ones [11]. Here, we explore those issues in the city of Paris (France), which has recently experienced a strong growth of its honey bee populations within a few years. In 2013, Paris hosted around 300 honey bee colonies, and in 2015 this figure had more than doubled, reaching 687 colonies, corresponding to 6.5 colonies.km^-2^ (data of the veterinary services of Paris; Fig 1), and has continued to increase since. In this context, our first objective was to analyze the effect of increasing honey bee colony densities on the visitation rates of wild pollinators at the community and morphological group levels. Secondly, we explored how the evenness of plant-pollinator networks was affected by increasing honey bee colony densities. The evenness index of plant-pollinator network reflects how balanced are the links realized by pollinators on plant communities. We expected that the interaction evenness of networks decreases along the gradient of increasing honey bee colony densities. Finally, we investigated the floral preferences of wild and domesticated pollinators for managed or wild plant species.

## Methods

### Study sites and plant-pollinator survey

**Fig 2:**
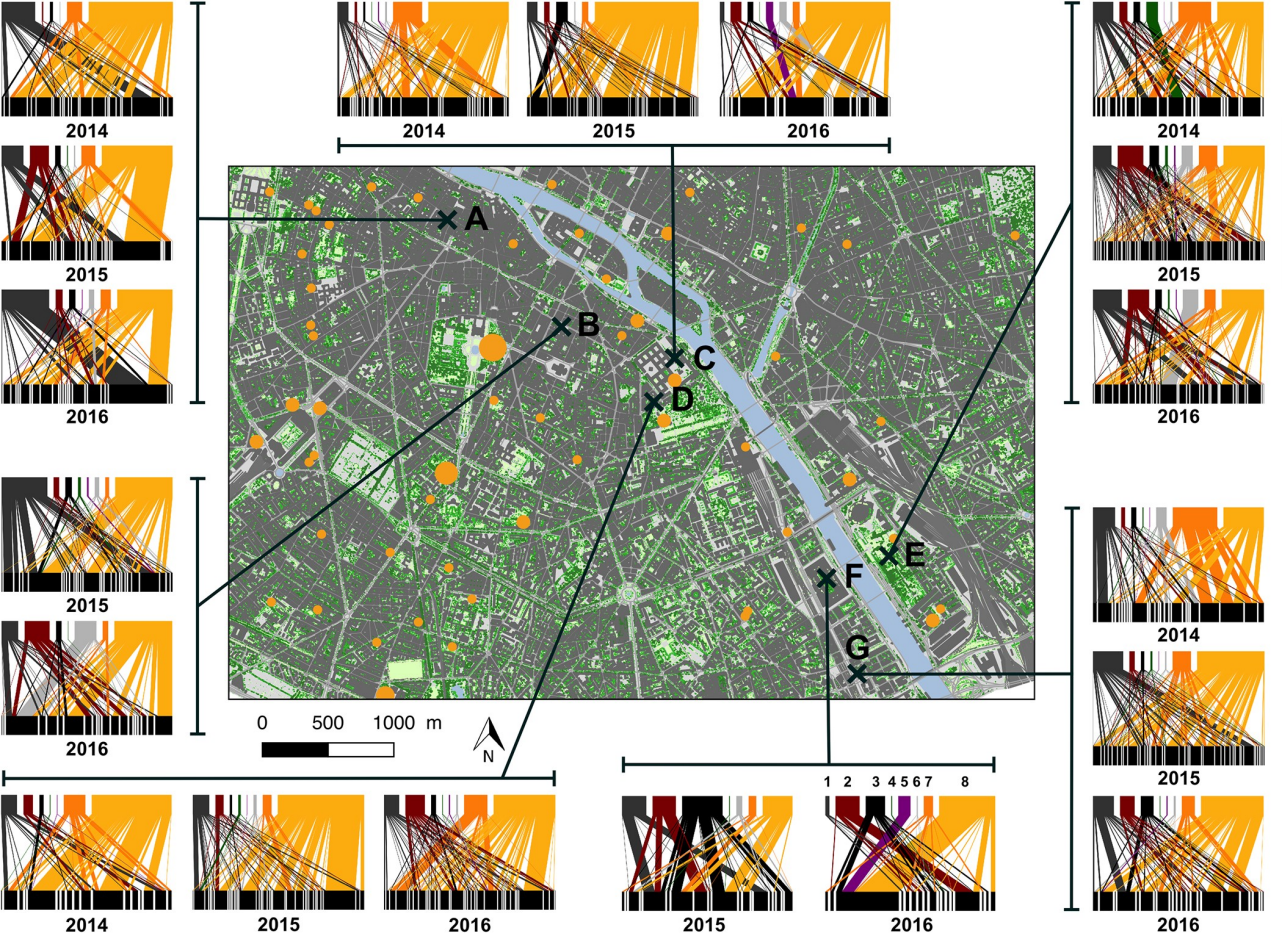
Study sites within Paris (France) and related plant-pollinator networks. Bipartite networks are retrieved from the compilation of all observed interactions between pollinators (top bar) and plants (bottom bar). Numbers under networks indicate the year. Each pollinator block represents a morphological group and each plant block represents a species. The width of links is proportional to the number of interactions (*i*.*e*. pollinating activity). Pollinator morphological groups are classified by order (left to right) colours and numbers: dark grey-1, small solitary bees; dark red-2, large solitary bees; black-3, syrphids; dark green-4, beetles; purple-5, butterflies; flies-6, light grey; orange-7, bumblebees; yellow-8, honey bees. Sites were represented by a capital letter to make the correspondance to S1 Data. Vegetation height and land use maps were obtained from APUR database (http://opendata.apur.org/datasets/).

The city of Paris (48°51′12″ N, 2°20′55″ E, Île-de-France, France) is a densely populated urban area (2 220 445 inhabitants in 2014, 105km^2^). In this city, for three consecutive years, we monitored plant-pollinator interactions in five (in 2014) to seven (in 2015 and 2016) green spaces. We chose these green spaces by their contrasted densities of honey bee colonies in their surroundings (Figs 1 and 2, S1 Data) and for their relative accessibility (access granted by the Bibliothèque nationale de France, campus of Paris Diderot University, Pierre et Marie Curie University, Descartes University, the Institut de Physique du Globe de Paris, and 2 gardens monitored by the Paris Direction des Espaces Verts et de l’Environnement). The distance between sites ranges from 410 to 6 264 meters (S2 Table). Honey bee colony densities were comprised between 0 and 28 colonies within 500m buffers around sites and between 7 and 53 colonies within 1000m buffers around sites (S1 Data). We chose to use the number of honey bee colonies around sites as it has been previously reported to be a good proxy to study potential competitive pressure exerted by honey bees on the wild pollinating fauna [11,18,21,22]. From May to July 2014 and from April to July 2015 and 2016, we carried 8, 11 and 13 observation rounds per green space respectively, spaced out at least by a week. For each round, in each site, we focused our observations on three one-meter square patches chosen to be the most flourished patches within flowerbeds. For each flower visited, we identified the visited plant to the lowest possible taxonomical level (from genus to horticultural variety) according to our knowledge and the taxonomic repository of France [23] and we classified it as managed or wild (S1 Table). Mean richness of visited plant species within patches could vary from 2.5 to 6.5 species depending on the flowering phenology of the plants present in the site. On each patch, we counted the number of visits realized by insect visitors during 5 minutes in 2014 and 2015 and 10 minutes in 2016. Each insect visitor was classified into one of these eight morphological groups: small and large solitary bees, honey bees–*A*. *mellifera*, bumblebees, beetles, butterflies, hoverflies and other flies [24]. Observation rounds were performed during warm sunny days (<15°C) with no wind and were carried out between 9 a.m. and 7 p.m. Because of daily variations in meteorological conditions, we alternated our samplings among sites between the morning and the afternoon from one week to the next.

### Location of honey bee colonies

The law requires beekeepers to report their honey bee colonies to the veterinary services of the city. This is to our knowledge the most accurate data existing to date regarding the location of honey bee colonies within Paris—even if we are aware that some beekeepers may not report their colonies. We used these data to estimate honey bee colony densities within 500- and 1000-meter buffers centred on the study sites using the ArcGIS software (Version 10.2). We chose these buffer sizes to match the mean foraging distances of the majority of wild and domesticated bee species [25,26].

### Statistical analyses

#### Spatial auto-correlation analysis

All statistical analyses were performed using R 3.2.2 (R Development Core Team, 2015). We checked the absence of spatial autocorrelation between our sites and honey bee colonies. We generated a matrix of distances between sites (S2 Table), and built matrices using the number of honey bee colonies in 500m and 1000m around our sites. Mantel tests were carried out between these matrices. No significant spatial autocorrelation was observed for both buffer sizes (500m –p = 0.749; 1000m –p = 0.204, respectively). We also tested the spatial autocorrelation of wild pollinator visitation rates. We found that the visitation rate of all wild pollinators together was spatially autocorrelated (p = 0.025) but not the morphological groups taken separately. Therefore, when analysing the visitation rate of all wild pollinators, we added an autoregressive process of order 1 correlation structure (addition of site coordinates and a random effect with sites nested within years) to deal with spatial autocorrelation.

#### Floral resources

Floral resources can affect the composition and activity of pollinator assemblages [27]. To account for the spatial heterogeneity of floral resources availability surrounding the study sites, we combined the area covered by the herbaceous, shrub and tree strata with an estimation of the average production of floral units per stratum along the observation period from February to July. We used a map of vegetation height, with a 50cm^2^/pixel resolution provided by the Parisian Urbanism Agency (APUR - http://opendata.apur.org/datasets/hauteur-vegetation-2015) to calculate the area covered by vegetation of less than 1 meter high (herbaceous stratum), between 1 and 10 meters (shrub stratum), and higher than 10 meters (tree stratum–Fig 1), this within buffers of 500 and 1000 meters centred on our study sites using Geographic Information Systems (GIS, ESRI ARC INFO v. 10.0). To estimate the floral resource production for shrubs and trees along the 6 months, we multiplied their area by their number of floral units/m^2^. The AgriLand database [28] (S3 Table) allowed us to estimate the number of floral units/m^2^ at a flowering peak. For these two strata, we considered that the flowering period lasted for 1 month [29]. For the herbaceous stratum, considering the flowering phenology, we modelled a normal distribution pattern (μ = 3; σ^2^ = 1.220) for 6 months, with the peak of floral production (2700 floral units/m^2^) occurring in the 3^rd^ month. Using this method, we averaged the number of floral units at 1,371 per month for the 6 months of flowering. Although not targeting urban areas, AgriLand database is the most comprehensive database on floral unit production, thus allowing to account for differences in floral resource production among vegetation stratum.

To assess the local floral resources, we calculated a mean richness of visited plant species by pollinators corresponding to the cumulated visited plant species per site per year divided by the number of observation rounds carried per site during the considered year.

#### Foraging activity analysis

To standardize the observation effort among years, we calculated visitation rates as the number of visits per minute and per flower visitor group on each site and for each year. Visitation rates were analysed using linear mixed effects models (lme, package “nlme”, R Development Core Team, 2015) and log transformed to approach normality.

Thus, for each morphological group, fixed effects were a) the honey bee colony densities at 500 or 1000 meters around our sites, b) the estimation of the floral resources available in buffers of the same radius and c) the mean visited plant species richness of each site. We included the sites nested within years as a random effect to account for temporal repetitions. We performed model simplification based on the Akaike Information Criterion (AIC) and chose the best fit model with ΔAIC > 2 (dredge, package “MuMIn”, R Development Core Team 2015 –S6 Table) [30] and then obtained the associate P-values using the Maximum Likelihood method (ML). All variables were scaled to make their estimated effects comparable.

We also checked the correlations between honey bee visitation rate and the visitation rate of other morphological groups. Fixed effects were the visitation rate of honey bees and the mean visited plant species richness; sites nested within years was added as a random effect. We selected the best fit model based on the Akaike Information Criterion (AIC) with a ΔAIC > 2 and then obtained the associate P-values using the Maximum Likelihood method (ML). We only detected that visitation rates of wild pollinators and bumblebees were significantly correlated to the local visited plant species richness. Once the mean richness of visited plants was taken into account in our models, we did not detect any significant relation between honey bee visitation rates and wild pollinator visitation rates all together or separated by morphological groups (S4 Table).

#### Plant-pollinator network analysis

To determine the impact of honey bee colony densities on the structure of plant-pollinator networks, we constructed 19 quantified interaction networks linking flower visitor morphological groups excluding honey bees to plant species, one per site and year. Interaction frequencies were calculated as the number of visits per minute. The structure of the interaction networks was assessed by the interaction evenness using the “bipartite” package [31]. Interaction evenness is bounded between 0 and 1, and derived from the Shannon index, *H* = p_ij_log_2_p_ij_/log_2_*F*, where *F* is the total number of plant–pollinator interactions in the matrix and p_ij_is the proportion of those interactions involving plant *i* and pollinator *j* [32,33]. This index reflects how balanced are the interaction strengths between plants and pollinators. It decreases as the network is dominated by few highly frequent interactions and increases when the number of interactions is uniformly distributed [34]. We analyzed the interaction evenness using the same statistical models than for the visitation rate analysis, fixed effects were a) the honey bee colony densities at 500 or 1000 meters around sites, b) the estimation of the floral resources available in a buffer of the same radius and c) the mean plant species richness of each site. A model simplification based on the Δ_AIC_ > 2 was used (S7 Table) [30] and the associated P-values were obtained using the Maximum Likelihood method (ML). We included the year nested within sites as a random effect to account for temporal repetitions.

#### Floral preferences analysis

To assess the pollinator floral preferences of both wild pollinators and honey bees, we summed their visitations on managed or wild plant species per site and per year (S1 Table). To consider the respective availability of both plant types, visitation rates of pollinator groups on managed or wild plants species were weighted by the percentage of managed and wild species recorded at each site and year. The number of each plant type sampled per year is available in the S5 Table. Floral preferences were tested using Student t-tests comparing the visitation rates of pollinator groups between managed and wild plants.

## Results

### Effect of honey bee colony densities on wild pollinator visitation rates

**Fig 3:**
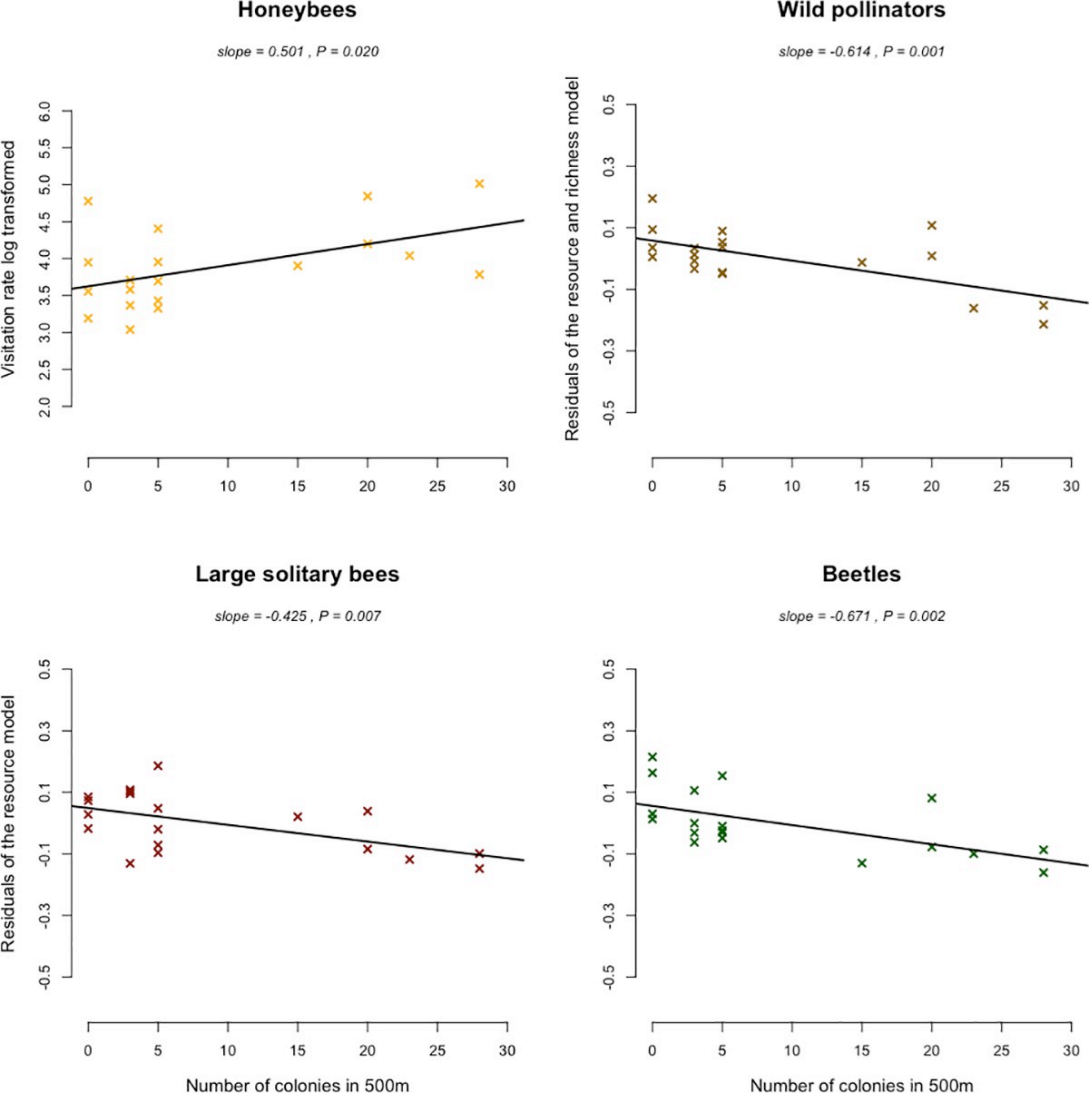
Morphological group visitation rates along the gradient of honey bee colony number in 500m around our observation sites. Regressions of best-fit models were represented for each morphological group. When best-fit models included multiple explanatory variables, partial residual regressions were plotted.

**Fig 4:**
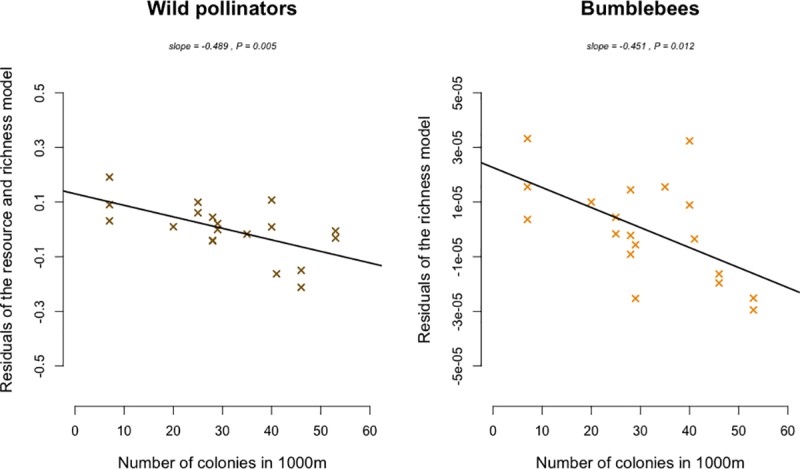
Morphological group visitation rates along the gradient of honey bee colony number in 1000m around our observation sites. Regressions of best-fit models were represented for each morphological group. When best-fit models included multiple explanatory variables, partial residual regressions were plotted.

Pollinators were monitored for a total of 3,120 minutes during which we recorded 795 individual plant-pollinator links, totalling 32,694 visits on plants (16% of small solitary bees, 10% of large solitary bees, 12% of bumblebees, 1% of beetles, 6% of hoverflies, 4% of flies, 1% of butterflies and 50% of honey bees). 687 honey bee colonies were declared in Paris in 2015, which equates to an average density of 6.5 colonies/km^2^. Visitation rates of wild pollinators were negatively related to the density of honey bee colonies at both spatial scales (Figs 3 and 4, and Table 1, 500m –slope = -0.614; p = 0.001 –and 1000m– slope = -0.489; p = 0.005). Large solitary bees performed significantly fewer visits when the density of honey bee colonies increased within 500 meter buffers around our observation sites (Fig 3, Table 1, slope = -0.425; p = 0.007). This trend was also significant for beetles (Fig 3, Table 1, slope = - 0.671; p = 0.002). The visitation rate of bumblebees significantly decreased when the density of honey bee colonies increased within 1000 meter buffers (Fig 4, Table 1, slope = - 0.451; p = 0.012). The visitation rate of honey bees was positively correlated with the number of honey bee colonies within 500 meter buffers (Fig 3, Table 1 –slope = 0.501; p = 0.020). However, we did not record any significant increase in the visitation rate of honey bees with the increased density of colonies within 1000 meter buffers. Finally, we did not find any effects of honey bee colony densities on the visitation rate of other morphological groups of pollinators such as small solitary bees, flies, hoverflies and butterflies (Δ_AIC_ < 2 between null models and convenience models or models containing only resources or richness variables–S6 and S7 Tables).

**Table 1:** Detailed effects of honey bee colony densities on wild pollinator visitation rates. Results of the best linear mixed-effects models containing the colonies number as response variable, and floral resources and richness, as covariables for each buffer scale. Model selection was performed according to the AIC criterion.

Morphological groups and scales	Predictor	Value	Standard deviation	Degree of freedom	t-value	P-value	AICc
Null model honey bees	Intercept	-0.008	0.272	16	-0.030	0.977	63.50
Honey bees 500m	Intercept	-0.012	0.274	15	-0.044	0.966	**61.00**
	Colonies number	0.501	0.193	15	2.597	0.020	
Null model wild pollinators	Intercept	-0.012	0.269	16	-0.045	0.964	63.50
Wild pollinators 500m	Intercept	-0.088	0.418	13	-0.210	0.837	**47.80**
	Colonies number	-0.614	0.133	13	-4.628	0.001	
	Resources	0.401	0.135	13	2.967	0.011	
	Richness	0.659	0.144	13	4.562	0.001	
Wild pollinators 1000m	Intercept	-0.083	0.416	13	-0.200	0.844	**56.30**
	Colonies number	-0.489	0.143	13	-3.408	0.005	
	Resources	0.186	0.142	13	1.316	0.211	
	Richness	0.527	0.175	13	3.008	0.010	
Null model large solitary bees	Intercept	-0.085	0.417	16	-0.203	0.842	59.70
Large solitary bees 500m	Intercept	-0.111	0.496	14	-0.224	0.826	**51.30**
	Colonies number	-0.425	0.133	14	-3.185	0.007	
	Resources	0.668	0.134	14	4.992	0.000	
Null model bumblebees	Intercept	0.017	0.267	16	0.062	0.952	63.80
Bumblebees 1000m	Intercept	0.000	0.147	14	0.000	1.000	**52.70**
	Colonies number	-0.451	0.155	14	-2.908	0.012	
	Richness	0.561	0.155	14	3.616	0.003	
Null model coleoptera	Intercept	-0.001	0.273	16	-0.004	0.997	63.50
Coleoptera 500m	Intercept	-0.012	0.285	14	-0.040	0.968	**56.60**
	Colonies number	-0.671	0.174	14	-3.854	0.002	
	Resources	0.714	0.175	14	4.092	0.001	

### Effect of honey bee colony densities on network structure

**Fig 5:**
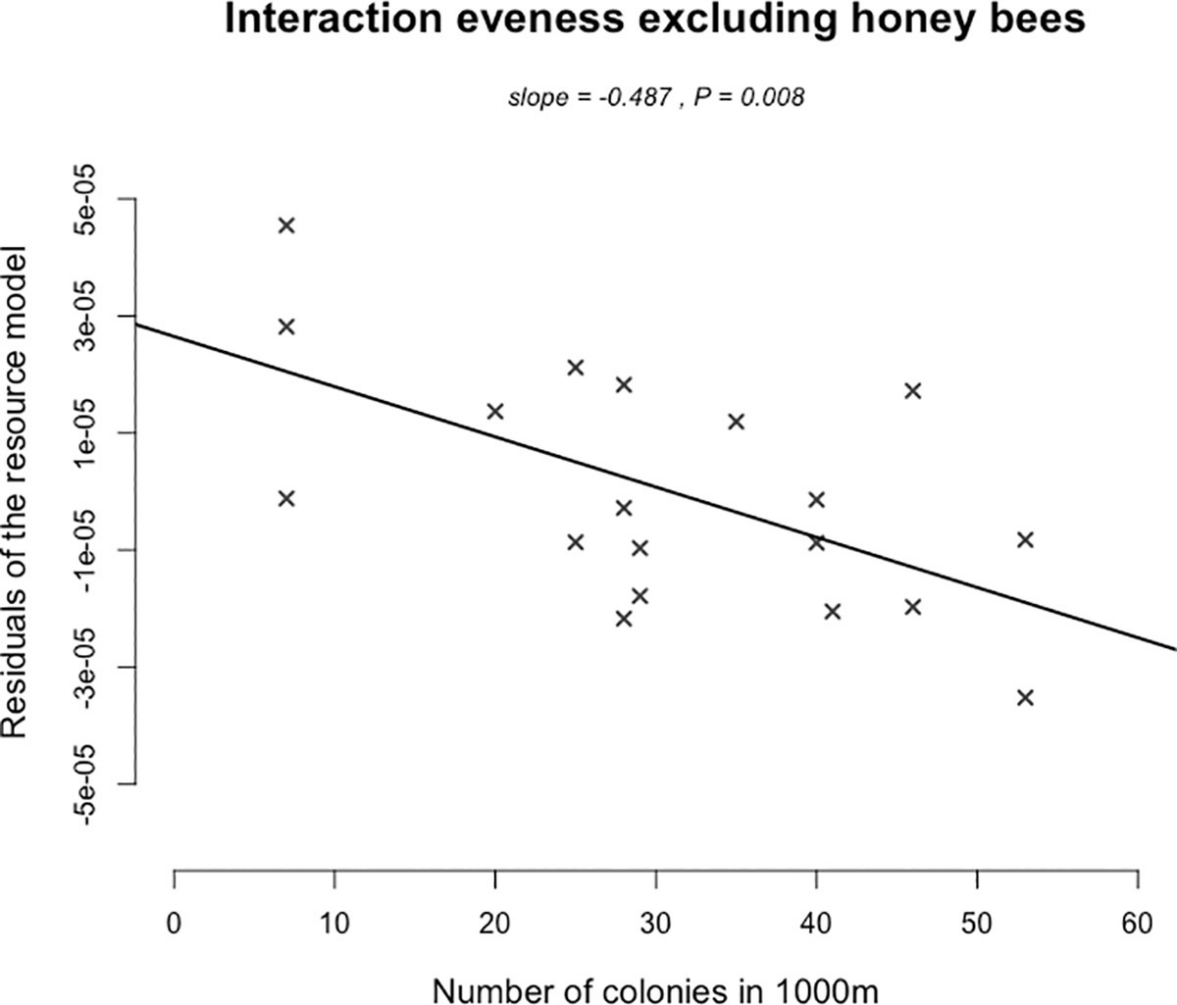
Partial regression of the interaction evenness along the gradient of increased honey bee colony numbers in 1000m buffers around our observation sites, once the effect of resources was taken into account.

Regarding the structure of the pollination networks, we found that the evenness of interactions between wild pollinators, excluding honey bees, and plants was negatively related to honey bee colony density within 1000 meter buffers (1000m –slope = -0.487; p = 0.008 –Fig 5, Table 2). We did not find any variation of the interaction evenness with the increased number of honey bee colonies within the 500 meter buffers. The model containing colony densities was equivalent to the null model (Δ_AIC_ < 2—S7 Table).

**Table 2:** Detailed effets of honey bee colony densities on the interaction evenness. Results of best linear mixed effects models on interaction eveness containing colonies number and floral resources as covariates. Model selection was performed according to the AIC criterion.

Network index	Predictor	Value	Standard deviation	Degree of freedom	t-value	P-value	AICc
Null model interaction evenness without honey bees	Intercept	0.000	0.229	16	0.000	1.000	63.70
Interaction evenness without honey bees 1000m	Intercept	0.000	0.153	14	0.000	1.000	**54.20**
	Colonies number	-0.487	0.159	14	-3.067	0.008	
	Resources	0.686	0.159	14	4.320	0.001	

### Floral preferences between wild and domesticated pollinators

**Fig 6:**
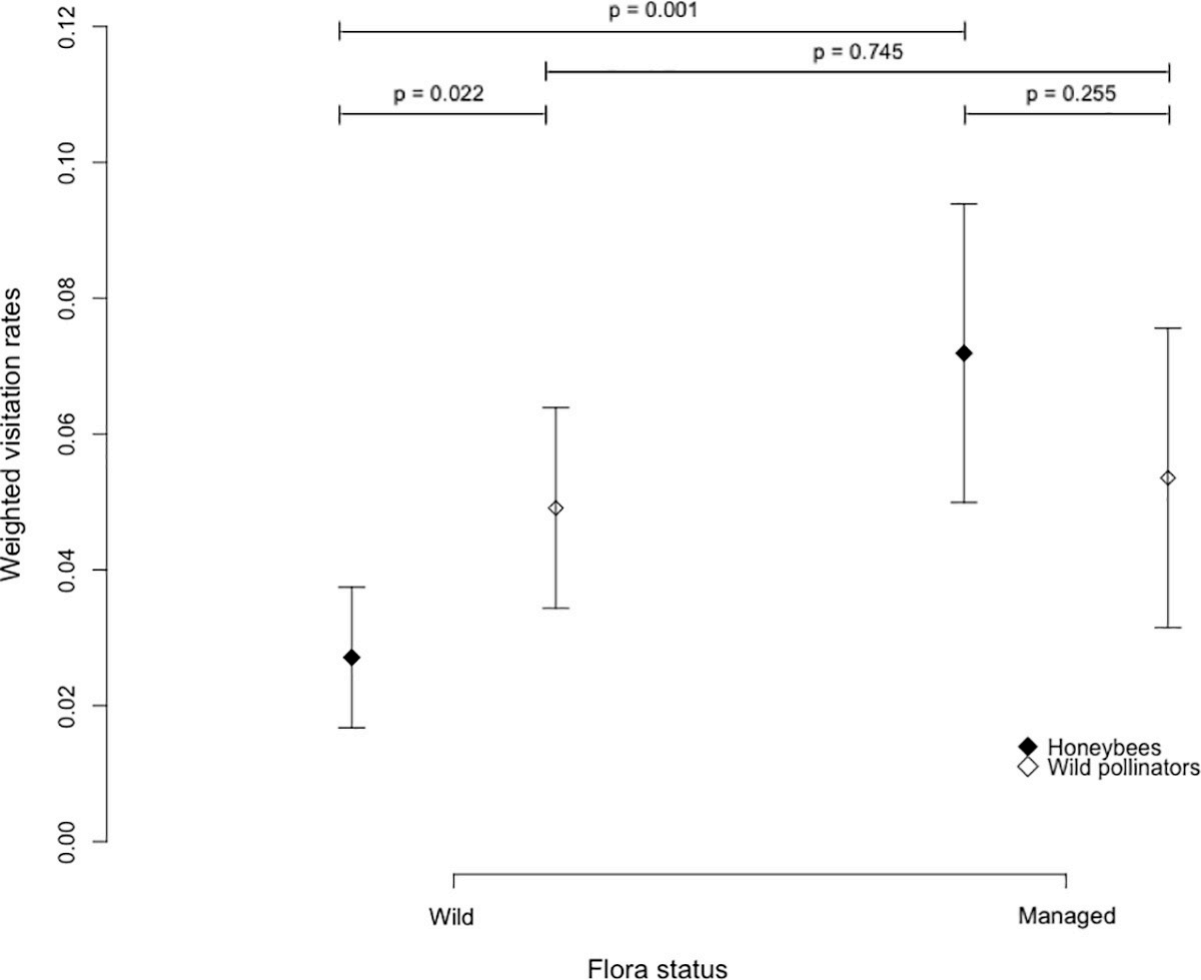
Floral preferences of honey bees and wild pollinators. Mean visitation rate on wild and managed flora weighted by the percentage of managed and wild plant species sampled at each site. Mean and 95% confidence intervals are represented. P-values were obtained with Student’s t-test.

We found that wild pollinators visited significantly more wild plant species than honey bees (t-test, p = 0.022). Furthermore, honey bees significantly preferred foraging on managed plant species than on wild ones (t-test, p = 0.001; Fig 6) whereas wild pollinators had no preference for a particular plant group, managed and wild plant species being equally visited (t-test, p = 0.745; Fig 6).

## Discussion

We showed that in the city of Paris, the visitation rate of wild pollinators and especially the pollinating activity of large solitary bees, bumblebees and beetles, was negatively related to the density of honey bee colonies in the surrounding landscape. This first finding resonates with a growing body of literature highlighting a negative effect of high honey bee colony densities on the wild pollinating fauna [11,21]. Although our study is correlative and does not provide direct evidences, our results are consistent with the hypothesis that honey bee might outcompete the wild pollinating fauna by exploiting flowering rewards (nectar and pollen) [18,19,35].

The negative correlation between the visitation rates of the total wild fauna and the honey bee colony density was found for both scales, within 500 and 1000 meter buffers. When focusing on each pollinator morphological group, this effect was however scale dependent. The visitation rate of large solitary bees and beetles was negatively correlated to honey bee colony density within 500 meter buffers whereas the visitation rate performed by honey bees increased. The bumblebee visitation rates were negatively correlated to the honey bee colony density within 1000 meter buffers. Those differences might be partly due to the foraging abilities of these groups. The large solitary bees includes numerous species which can forage from few hundred meters to several kilometers from their nest, depending on the species considered and the landscape context [25,36]. Bumblebees on the other hand are known to forage at large scales, up to 2800 meters from their nest [37]. Large solitary bees, bumblebees and honey bees have similar dietary requirements, exploiting the same floral resources (pollen and nectar) [17,38]. As summarized in Wojcik et al. 2018 [39], previous studies have found that adding honey bee colonies may negatively affect wild bees and particularly bumblebees especially due to this overlap in resource use.

On the contrary, flies, syrphids and butterflies do not exclusively rely on floral resources, especially during their larval life-stage, which might explain the absence of negative interactions with honey bees [40]. Small solitary bees do require pollen and nectar for both larva and adult stages but because body size and mouthparts length are correlated traits [41], small solitary bees might prefer to seek resources preferentially on shallow flowers [24]. Conversely, larger pollinators such as honey bees and bumblebees, could preferentially forage on plants best adapted to their morphology (preferentially deep flowers—see [42,43]). In that way, small solitary bees might be less sensitive to the increase in honey bee colony densities. The sharp decline of beetles’ foraging activity with the honey bee colony density within 500 meter buffers is more surprising. There is little literature on floral preferences of beetles. Also, their foraging range seems to be highly variable. As examples, Englund (1993) found that *Cetonia aurata* had a 18m foraging range and Juhel et al. (2017) estimated the foraging range of *Brassicogethes aeneus* up to 1.2km [44,45]. This underlines the difficulty to relate scale dependent ecological effects with ecological traits of species. For honey bees, we did not detect any increase in their visitation rate with honey bee colony density within 1000 meter buffers. Honey bee foraging range seems to be highly context dependent, from several hundred meters to several kilometers [46,47]. Additionally, Couvillon et al. 2015 demonstrated that honey bee foraging distances both depend on the type of rewards that honey bees seek (nectar or pollen) and on the month considered [48]. The scale to which organisms respond to landscape characteristics thus appear dependent of the context and sensitive to various components acting together. In dense urban habitats, pollinator’s foraging distance might also be sensitive to building height, width or to the spatial distribution of green spaces and floral resources [49].

We also recorded a decrease in the evenness of plant-pollinator interaction networks with the honey bee colony density within 1000 meter buffers. Interaction evenness decreases when the network is dominated by few and/or highly frequent interactions. A high evenness has been previously associated with a good network stability [50,51]. Being opposite, a low interaction evenness has been highlighted in degraded ecosystems [52] and in invaded networks [53]. In a previous meta-analysis [11], we showed that the honey bee position in interaction networks is comparable to that often found for invasive pollinators. Here, the lower evenness at high colonies densities within 1000 meter buffers could be due to the decrease of wild pollinators and particularly of bumblebee’s visitation rate. This questions the potential impact of urban beekeeping on the whole interaction network and urges once again the need for news studies regarding this topic.

In parallel, we showed that honey bees tended to significantly focus their visits on managed plant species, whereas wild pollinators did not show preferences between managed and wild plants. Honey bees often focus their visits on the most abundant resources to cover the colony needs [54] and ornamental flowerbeds might thus be attractive for them. Among the species most visited by honey bees, we indeed found *Lavandula sp*. and *Geranium sanguineum* which are common in ornamental flowerbeds. In the other hand, wild flowers received significantly more visits from wild pollinators and might rely more on the wild fauna for pollination. The observed decline of the wild fauna visitation rate associated with high colony densities may have negative consequences for the reproduction of this wild flora. Nevertheless, several other factors might explain insect’s flowers preferences and foraging choices such as the morphology, the color, the amount of resources, or the life span of flowers [55].

As this study took place in a city, urban environment may provide a large range of confounding factors such as pollutions, pesticides or floral resources quantity, which could also explain the observed decline in the foraging activity of some morphological groups. Few studies have explored the impact of pollutants on bees in urban areas, Moroń et al., 2012 & 2014 demonstrated that heavy metal pollution decreased the diversity and the abundance of solitary bees and can reduce the fitness of *Osmia rufa*, a common Megachilidae in urban environments [56,57]. Concerning pesticides, in Paris, public parks do not use any pesticides in their management since 2008, which limits the impact of this factor. Finally, we observed pollinators activity from April to July corresponding to the peak of floral resources, and consequently at this period there is a large amount of nectar and pollen available to flower visitors. However, along a year, the quantity of floral resources fluctuates and leads to peaks and gaps in floral resources abundance [58]. Here, we found a negative relation between wild pollinator foraging activities and honey bee colony densities but the intensity of this relation could be modulated by the amount of resources available that could be less abundant in early spring or late summer for example.

In this study, the proxy we did use to study the potential impact of honey bees on the wild fauna was the density of honey bee colonies around our sites, and this proxy has been used in several other studies [18,22]. We did not however find any significant correlations between the visitation rate of honey bees and the visitation rate of wild pollinators. We advocate here that the honey bee colony density variable represents a more continuous pressure on the wild fauna that simply the foraging activity of honey bees at a given time. However, at this stage, and as underlined by Mallinger et al. (2017), other critical parameters such as wild pollinator reproductive success (fitness), population or community dynamics are yet rarely explored [21]. This lack of knowledge impedes us to have a more comprehensive view of the potential impact of high honey bee colony densities on the wild pollinating fauna.

Nevertheless, numerous cities around the globe have experienced recent and fast increases in honey bee colony densities. The average colony density in Paris (6.5 colonies/km^2^) is higher than the national level (2.5 colonies/km^2^) but is far below other cities such as Brussels (15 colonies/km^2^) or London (10 colonies/km^2^) [13,14,59]) and cities also harbor a non-negligible diversity of wild pollinators [60–62]. Altogether, our results not only question the fast development of urban beekeeping and the enthusiasm of citizens and mass media for the installation of hives in cities, but also some of urban management practices supposedly conducted to sustain biodiversity. This underlines the need for new studies exploring how domestic and wild pollinators coexist in urban habitats. In conclusion, we suggest that stakeholders should take into account the impacts that apiaries could have on the wild fauna [19,35]. If the capacity of urban ecosystems to provide the pollination function is to be preserved, land owners may focus their management practices on increasing floral resources and nesting habitats for pollinators in urban environments instead of adding honey bee colonies.

## Supporting information

S1 Table List of plant species with their status. (DOCX)

S2 Table Distance between study sites in meters. (DOCX)

S3 Table Open floral unit number per m^2^ of vegetative cover at the peak of flowering season from AgriLand Database. (DOCX)

S4 Table Detailed effects of honey bee visitation rates on wild pollinator visitation rates. Results of the best linear mixed-effects models containing the visitation rates of honey bees as response variable and mean visited plant richness as covariable. Visitation rates were log transformed. Model selection was performed according to AIC criterion. Models with others morphological groups were equal to the null model and were not presented here (delta AIC < 2).
(DOCX)

S5 Table Number of plants occurrences sampled per year, according to their status (wild or managed species). (DOCX)

S6 Table Results of morphological groups’ model selection based on AIC criterion. We present here all the models with negative delta AIC from the null model.
(DOCX)

S7 Table Results of interaction evenness model selection based on AIC criterion. We present here all the models with negative delta AIC from the null model.
(DOCX)

S1 Data Dataset of the study. (CSV)
