# Supplementary material for: Wild pollinator activity negatively related to honey bee colony densities in urban context
Source: PLoS One. 2019 Sep 12;14(9):e0222316. doi: 10.1371/journal.pone.0222316 (PMC6742366; doi:10.1371/journal.pone.0222316)
Supplement: S2 Table — (DOCX) [file pone.0222316.s002.docx]

**S2 Table. Distance between study sites in meters.**

| Distance (m) | Site 1 | Site 2 | Site 3 | Site 4 | Site 5 | Site 6 |
| --- | --- | --- | --- | --- | --- | --- |
| Site 2 | 723.84 |  |  |  |  |  |
| Site 3 | 4238.60 | 3732.95 |  |  |  |  |
| Site 4 | 3066.09 | 2559.29 | 1176.64 |  |  |  |
| Site 5 | 5965.41 | 5459.33 | 1729.15 | 2905.76 |  |  |
| Site 6 | 2954.27 | 2528.07 | 1335.80 | 410.04 | 3032.67 |  |
| Site 7 | 881.36 | 846.97 | 4545.26 | 3379.50 | 6264.35 | 3369.72 |
