# Supplementary material for: Wild pollinator activity negatively related to honey bee colony densities in urban context
Source: PLoS One. 2019 Sep 12;14(9):e0222316. doi: 10.1371/journal.pone.0222316 (PMC6742366; doi:10.1371/journal.pone.0222316)
Supplement: S3 Table — (DOCX) [file pone.0222316.s003.docx]

**S3 Table. Open floral unit number per m² of vegetative cover at the peak of flowering season from AgriLand Database.**

| Strata | Floral density at the flowering peak |
| --- | --- |
| Small (max height <1 m) | 2713.6 |
| Medium (1 m ≤ max height <10 m) | 2186.4 |
| Tall (max height ≥ 10 m) | 5291.4 |
