# Supplementary material for: Wild pollinator activity negatively related to honey bee colony densities in urban context
Source: PLoS One. 2019 Sep 12;14(9):e0222316. doi: 10.1371/journal.pone.0222316 (PMC6742366; doi:10.1371/journal.pone.0222316)
Supplement: S4 Table — Results of the best linear mixed-effects models containing the visitation rates of honey bees as response variable and mean visited plant richness as covariable. Visitation rates were log transformed. Model selection was performed according to AIC criterion. Models with others morphological groups were equal to the null model and were not presented here (delta AIC < 2). (DOCX) [file pone.0222316.s004.docx]

**S4 Table. Detailed effects of honey bee visitation rates on wild pollinator visitation rates.** Results of the best linear mixed-effects models containing the visitation rates of honey bees as response variable and mean visited plant richness as covariable. Visitation rates were log transformed. Model selection was performed according to AIC criterion. Models with others morphological groups were equal to the null model and were not presented here (delta AIC < 2).

| **Visitation rates of morphogroups** | **Predictor** | **Value** | **Standard deviation** | **Degree of freedom** | **t-value** | **P-value** | **AICc** |
| --- | --- | --- | --- | --- | --- | --- | --- |
| Null model wild pollinators | Intercept | 3.898 | 0.121 | 16 | 32.099 | NA | 30.49 |
| Wild pollinators | Intercept | 2.881 | 0.372 | 15 | 7.740 | 0.000 | **25.44** |
|  | **Plant richness** | 0.249 | 0.083 | 15 | 2.989 | 0.009 |  |
| Null model bumblebees | Intercept | 2.449 | 0.202 | 16 | 12.096 | NA | 50.15 |
| Bumblebees | Intercept | 0.687 | 0.495 | 15 | 1.388 | 0.185 | **41.23** |
|  | **Plant richness** | 0.438 | 0.119 | 15 | 3.671 | 0.002 |  |
