# Supplementary material for: Wild pollinator activity negatively related to honey bee colony densities in urban context
Source: PLoS One. 2019 Sep 12;14(9):e0222316. doi: 10.1371/journal.pone.0222316 (PMC6742366; doi:10.1371/journal.pone.0222316)
Supplement: S5 Table — (DOCX) [file pone.0222316.s005.docx]

**S5 Table. Number of plants occurrences sampled per year, according to their status (wild or managed species).**

| **Year** | **Number of wild plant occurrences sampled for all observation round** | **Number of managed plant occurrences sampled for all observation round** |
| --- | --- | --- |
| 2014 | 106 | 72 |
| 2015 | 148 | 218 |
| 2016 | 116 | 119 |
| Total | 370 | 409 |
