# Supplementary material for: Wild pollinator activity negatively related to honey bee colony densities in urban context
Source: PLoS One. 2019 Sep 12;14(9):e0222316. doi: 10.1371/journal.pone.0222316 (PMC6742366; doi:10.1371/journal.pone.0222316)
Supplement: S6 Table — We present here all the models with negative delta AIC from the null model. (DOCX) [file pone.0222316.s006.docx]

**S6 Table. Results of morphological groups’ model selection based on AIC criterion.** We present here all the models with negative delta AIC from the null model.

| **Morphogroups and scales** | |  | | **Intercept** | **Colonies** | **Resources** | **Mean Richness** | **Df** | **log Likelihood** | **AICc** | **Delta** | **Weight** |
| --- | --- | --- | --- | --- | --- | --- | --- | --- | --- | --- | --- | --- |
| Honey bees 500m | | 2 | | -0.012 | 0.501 |  |  | 5 | -23.207 | 61.00 | 0.00 | 0.544 |
|  | | Null model | | -0.008 |  |  |  | 4 | -26.311 | 63.50 | 2.45 | 0.160 |
| Honey bees 1000m | | Null model | | 3.878 |  |  |  | 4 | -15.305 | 41.50 | 0.00 | 0.546 |
| Wild pollinators 500m | | 8 | | -0.088 | -0.614 | 0.401 | 0.659 | 7 | -14.933 | 54.00 | 0.00 | 0.782 |
|  | | 6 | | -0.079 | -0.414 |  | 0.710 | 6 | -19.126 | 57.30 | 3.20 | 0.158 |
|  | | 5 | | -0.062 |  |  | 0.634 | 5 | -22.816 | 60.20 | 6.20 | 0.035 |
|  | | 4 | | -0.033 | -0.622 | 0.497 |  | 6 | -22.138 | 63.30 | 9.23 | 0.008 |
|  | | Null model | | -0.012 |  |  |  | 4 | -26.341 | 63.50 | 9.49 | 0.007 |
| Wild pollinators 1000m | | 6 | | -0.084 | -0.450 | 0.581 |  | 6 | -18.645 | 56.30 | 0.00 | 0.644 |
|  | | 8 | | -0.083 | -0.489 | 0.186 | 0.527 | 7 | -17.611 | 59.40 | 3.11 | 0.136 |
|  | | 5 | | -0.062 |  |  | 0.634 | 5 | -22.816 | 60.20 | 3.96 | 0.089 |
|  | | 2 | | -0.038 | -0.522 |  |  | 5 | -23.104 | 60.80 | 4.53 | 0.067 |
|  | | 4 | | -0.043 | -0.573 | 0.292 |  | 6 | -21.644 | 62.30 | 6.00 | 0.032 |
|  | | Null model | | -0.012 |  |  |  | 4 | -26.341 | 63.50 | 7.25 | 0.017 |
| Small solitary bees 500m | | 2 | | -0.011 | -0.400 |  |  | 5 | -24.125 | 62.90 | 0.00 | 0.375 |
|  | | Null model | | -0.008 |  |  |  | 4 | -26.089 | 63.00 | 0.17 | 0.345 |
| Small solitary bees 1000m | | Null model | | -0.008 |  |  |  | 4 | -26.089 | 63.00 | 0.00 | 0.347 |
| Large solitary bees 500m | | 4 | | -0.111 | -0.425 | 0.668 |  | 6 | -16.163 | 51.30 | 0.00 | 0.753 |
|  | | 8 | | -0.125 | -0.422 | 0.641 | 0.178 | 7 | -15.278 | 54.70 | 3.41 | 0.137 |
|  | | 3 | | -0.101 |  | 0.450 |  | 5 | -20.594 | 55.80 | 4.48 | 0.08 |
|  | | 7 | | -0.114 |  | 0.427 | 0.164 | 6 | -20.188 | 59.40 | 8.05 | 0.013 |
|  | | Null model | | -0.085 |  |  |  | 4 | -24.444 | 59.70 | 8.42 | 0.011 |
| Large solitary bees 1000m | | 3 | | -0.094 |  | 0.466 |  | 5 | -20.173 | 55.00 | 0.00 | 0.498 |
|  | | 4 | | -0.106 | -0.256 | 0.505 |  | 6 | -18.391 | 55.80 | 0.82 | 0.33 |
|  | | 7 | | -0.106 |  | 0.442 | 0.138 | 6 | -19.882 | 58.80 | 3.80 | 0.074 |
|  | | Null model | | -0.085 |  |  |  | 4 | -24.444 | 59.70 | 4.78 | 0.046 |
| Bumblebees 500m | | 5 | | 0.000 |  |  | 0.665 | 5 | -20.901 | 56.40 | 0.00 | 0.674 |
|  | | 7 | | 0.000 |  | 0.228 | 0.622 | 6 | -20.008 | 59.00 | 2.60 | 0.184 |
|  | | 6 | | 0.000 | -0.059 |  | 0.668 | 6 | -20.84 | 60.70 | 4.26 | 0.08 |
|  | | 8 | | 0.000 | -0.229 | 0.344 | 0.611 | 7 | -19.245 | 62.70 | 6.25 | 0.03 |
|  | | Null model | | 0.017 |  |  |  | 4 | -26.363 | 63.60 | 7.17 | 0.019 |
| Bumblebees 1000m | | 6 | | 0.000 | -0.451 |  | 0.561 | 6 | -16.87 | 52.70 | 0.00 | 0.614 |
|  | 8 | | 0.000 | | -0.502 | 0.247 | 0.506 | 7 | -15.267 | 54.70 | 1.98 | 0.229 |
|  | 5 | | 0.000 | |  |  | 0.665 | 5 | -20.901 | 56.40 | 3.68 | 0.098 |
|  | 2 | | 0.012 | | -0.565 |  |  | 5 | -22.448 | 59.50 | 6.77 | 0.021 |
|  | 4 | | 0.017 | | -0.612 | 0.353 |  | 6 | -20.323 | 59.60 | 6.91 | 0.019 |
|  | 7 | | 0.000 | |  | 0.147 | 0.639 | 6 | -20.537 | 60.10 | 7.33 | 0.016 |
|  | Null model | | 0.017 | |  |  |  | 4 | -26.363 | 63.60 | 10.84 | 0.003 |
| Coleoptera 500m | 4 | | -0.012 | | -0.671 | 0.714 |  | 6 | -18.778 | 56.60 | 0.00 | 0.83 |
|  | 8 | | -0.018 | | -0.665 | 0.686 | 0.154 | 7 | -18.352 | 60.90 | 4.33 | 0.095 |
|  | Null model | | -0.001 | |  |  |  | 4 | -26.299 | 63.50 | 6.90 | 0.026 |
| Coleoptera 1000m | Null model | | -0.001 | |  |  |  | 4 | -26.299 | 63.50 | 0.00 | 0.432 |
| Lepidoptera 500m | Null model | | -0.010 | |  |  |  | 4 | -26.392 | 63.60 | 0.00 | 0.626 |
| Lepidoptera 1000m | Null model | | -0.010 | |  |  |  | 4 | -26.392 | 63.60 | 0.00 | 0.565 |
| Syrphids 500m | Null model | | -0.041 | |  |  |  | 4 | -26.001 | 62.90 | 0.00 | 0.506 |
| Syrphids 1000m | Null model | | -0.041 | |  |  |  | 4 | -26.001 | 62.90 | 0.00 | 0.38 |
| Diptera 500m | 5 | | -0.077 | |  |  | 0.664 | 5 | -24.289 | 63.20 | 0.00 | 0.404 |
|  | Null model | | 0.000 | |  |  |  | 4 | -26.446 | 63.70 | 0.56 | 0.306 |
| Diptera 1000m | 5 | | -0.077 | |  |  | 0.664 | 5 | -24.289 | 63.20 | 0.00 | 0.445 |
|  | Null model | | 0.000 | |  |  |  | 4 | -26.446 | 63.70 | 0.56 | 0.337 |
