# Supplementary material for: Wild pollinator activity negatively related to honey bee colony densities in urban context
Source: PLoS One. 2019 Sep 12;14(9):e0222316. doi: 10.1371/journal.pone.0222316 (PMC6742366; doi:10.1371/journal.pone.0222316)
Supplement: S7 Table — We present here all the models with negative delta AIC from the null model. (DOCX) [file pone.0222316.s007.docx]

**S7 Table. Results of interaction evenness model selection based on AIC criterion.** We present here all the models with negative delta AIC from the null model.

| **Network index and scale** |  | **Intercept** | **Colonies** | **Resources** | **Mean Richness** | **Df** | **log Likelihood** | **AICc** | **Delta** | **Weight** |
| --- | --- | --- | --- | --- | --- | --- | --- | --- | --- | --- |
| Interaction evenness without | 3 | 0.000 |  | 0.590 |  | 5 | -22.375 | 59.40 | 0.00 | 0.663 |
| honey bees 500m | 4 | 0.000 | -0.238 | 0.709 |  | 6 | -21.735 | 62.50 | 3.10 | 0.141 |
|  | 7 | 0.000 |  | 0.576 | 0.076 | 6 | -22.294 | 63.60 | 4.22 | 0.080 |
|  | Null model | 0.000 |  |  |  | 4 | -26.446 | 63.70 | 4.38 | 0.074 |
| Interaction evenness without | 4 | 0.000 | -0.487 | 0.686 |  | 6 | -17.599 | 54.20 | 0.00 | 0.819 |
| honey bees 1000m | 3 | 0.000 |  | 0.612 |  | 5 | -21.993 | 58.60 | 4.40 | 0.091 |
|  | 8 | 0.000 | -0.501 | 0.698 | -0.054 | 7 | -17.537 | 59.30 | 5.06 | 0.065 |
|  | 7 | 0.000 |  | 0.598 | 0.079 | 6 | -21.900 | 62.80 | 8.60 | 0.011 |
|  | Null model | 0.000 |  |  |  | 4 | -26.446 | 63.70 | 9.55 | 0.007 |
